# Positive and negative syndrome scale in forensic patients with schizophrenia spectrum disorders: a systematic review and meta-analysis

**DOI:** 10.1186/s12991-022-00413-2

**Published:** 2022-09-10

**Authors:** Chiara Buizza, Cosmo Strozza, Giulio Sbravati, Giovanni de Girolamo, Clarissa Ferrari, Laura Iozzino, Ambra Macis, Harry G. Kennedy, Valentina Candini

**Affiliations:** 1 Psychiatric Epidemiology and Evaluation Unit, IRCCS Istituto Centro San Giovanni Di Dio Fatebenefratelli, Via Pilastroni 4, 25125 Brescia, Italy; 2 Department of Clinical and Experimental Sciences, University of Brescia, Viale Europa 11, 25123 Brescia, Italy; 3 Interdisciplinary Centre On Population Dynamics, University of Southern Denmark, 5000 Odense, Denmark; 4 Service of Statistics, IRCCS Istituto Centro San Giovanni Di Dio Fatebenefratelli, Via Pilastroni 4, Brescia, Italy; 5 The National Forensic Mental Health Service, Central Mental Hospital, Dundrum, Dublin 14, Ireland; 6 Academic Department of Psychiatry, Trinity College Dublin, Dublin, Ireland

**Keywords:** Forensic patient, Schizophrenia spectrum disorders, Symptomatology, PANSS, Psychotic symptoms

## Abstract

Among forensic patients with schizophrenia spectrum disorders, the association between symptomatology and violence is still not entirely clear in literature, especially because symptoms shift both during the acute phase of the illness and after. The aims were to investigate the level of symptomatology in forensic patients and to evaluate if there are differences in the level of symptoms between forensic and non-forensic patients. According to PRISMA guidelines, a systematic search was performed in PubMed, Web of Science, and ProQuest, using the following key words: “forensic” AND “Positive and Negative Syndrome Scale” OR “PANSS”. A total of 27 studies were included in the systematic review, while only 23 studies in the meta-analysis. The overall sample included a total of 1702 participants, most commonly male and inpatients in forensic settings. We found that studies with an entirely male sample had significantly lower Positive PANSS ratings than studies with mixed samples. Although both forensic and non-forensic patients were affected by mild psychopathological symptoms, forensic patients presented higher ratings in all four PANSS scales. This meta-analysis shows that forensic patients reported a mild level of symptomatology, as assessed with the PANSS, and therefore might be considered as patients in partial remission. Among patients with schizophrenia, the association between symptoms and violence is very complex: many factors might be considered as key mediators and thus should be taken into account to explain this association. Further studies are needed.

*Trial registration* all materials and data can be found on the OSF framework: https://osf.io/5ceja (date of registration: 8 September 2021)

**Supplementary Information:**

The online version contains supplementary material available at 10.1186/s12991-022-00413-2.

## Background

Many studies on the association between schizophrenia and violence have been published in recent years [1–4]. These studies showed that violent behaviour is more common during the acute phase of the illness [4–9]. A meta-analysis of 110 studies on risk factors for violence in psychosis concluded that higher general symptom ratings and higher total Positive and Negative Syndrome Scale (PANSS [10]) ratings were associated with violence. With regards to specific positive symptoms, violence was associated with higher excitement ratings and higher positive symptoms ratings. According to one meta-analysis, violence was not significantly associated with negative symptomatology [11], while others found that negative symptoms were inversely associated with the risk of violence [12–14].

Despite these results, the role played by psychotic symptoms is still not entirely clear in literature, especially because symptoms shift both during the acute phase and later in the course of this disorder [13]. For this reason, it seems important to understand what are the symptom profiles of people at a different time to when they committed violence, particularly in people who have committed crimes and who live in forensic services. Forensic patients are a heterogeneous population who differ widely in diagnoses, crimes committed, and risk factors. All these aspects are important, have treatment implications, and should consequently be accounted for in research. Violent acts are also diverse, whether minimal or homicidal in severity, whether deliberative or impulsive, instrumental or expressive, intoxicated, driven by delusions, or in the absence of an abnormal mental state [15–17].

Given the wide interest for the structured clinical assessment of severe mental disorders and the large utilization of PANSS to evaluate symptomatology in patients with Schizophrenia Spectrum Disorders (SSDs), we decided to conduct a systematic review and meta-analysis on PANSS ratings in forensic populations.

## Methods

The aims of this systematic review and meta-analysis were (a) to investigate the level of psychotic symptomatology assessed by the PANSS in patients with SSDs in treatment at psychiatric forensic institutions and to identify specific variables associated with symptom severity and (b) to assess differences in symptom severity between forensic and non-forensic patients with SSDs.

The PANSS [10] is a 30-item rating scale on a scale of 1 (absent) to 7 (severe) developed by combining the 18 items of the Brief Psychiatric Rating Scale (BPRS) [18] and 12 items from the Psychopathology Rating Scale (PRS) using clear definitions to tether each gradation of each item; the PANSS overall total score ranges from 30 to 210.

### Protocol and registration

In accordance with the Preferred Reporting Items for Systematic Reviews and Meta-Analyses (PRISMA) guidelines [19], our systematic review protocol was registered with Open Science Foundation (OSF) database. All materials and data can be found on the OSF framework: https://osf.io/5ceja (date of registration: 8 September 2021).

### Eligibility criteria

To be included in the meta-analysis, we defined the following eligibility criteria: (a) studies focusing on adult forensic patients (age  > 18 years); (b) studies, including patients suffering from SSDs (at least 50% of the sample); and (c) studies reporting PANSS mean ratings. Since studies on forensic populations are in limited number, we included studies with different designs (randomized clinical trials, observational studies). All studies included patients with mental illness who committed any offences and therefore were in charge of forensic services (including outpatients and inpatients). We excluded case reports, dissertations, protocols, reviews, case series studies, unpublished studies, and studies in languages other than English.

### Information sources and search strategy

All published peer-reviewed articles were retrieved through a systematic literature search on PubMed, Web of Science and ProQuest from inception to September 2020, using the following key words: “forensic” AND “Positive and Negative Syndrome Scale” OR “PANSS”.

### Study selection

Two authors independently (CB and GS) screened the article titles and abstracts for inclusion and exclusion criteria and extracted data from all full-text articles selected. Any disagreements in data extraction process were negotiated among two authors.

### Data collection process

Data were collected in a specific data extraction form, reporting the following items: (a) study characteristics––authorship, year, country of recruitment, and study design; (b) sample characteristics––number of subjects with SSDs who entered the study, mean age, gender, type of treatment (inpatients or outpatients), illness duration, and length of stay; and (c) PANSS mean ratings available (Positive, Negative, General, and Total). When the studies included non-forensic patients, we also collected their data. Moreover, for studies reporting PANSS mean ratings at baseline and follow-up, only baseline ratings were used, in order to exclude the effect of specific treatment on PANSS scores.

### Statistical analysis

Data analysis was divided into two parts: the first only included forensic patients, while the second comprised both forensic and non-forensic patients.

Firstly, random-effects meta-analysis was performed to calculate a pooled estimate of the mean PANSS rating for each scale (Positive, Negative, General, and Total) with 95% confidence intervals among forensic patients. We decided for a random-effects approach assuming there would be heterogeneity among the studies included in the analysis [20]. Some characteristics of studies were identified as potential modifiers of the reported results. For this reason, subgroup analysis was performed in order to evaluate the differences in PANSS mean ratings (four scales) among studies with: SSD patients only or also including non-SSD patients; inpatient (In), or outpatient study setting (Out, Mix); males only or also including females; patients older than the first quartile of the age distribution or patients within the first quartile; and high-quality or low-quality studies.

Secondly, random-effects meta-analysis was performed to calculate a pooled estimate of the mean difference between forensic and non-forensic patients in each of the PANSS scales (Positive, Negative, General, and Total rating) with 95% confidence intervals.

In order to measure the heterogeneity among the studies included in the analysis, we used the *Q*-statistic and *I*^2^ index (% of total variability due to heterogeneity): a significant value of *Q* and an *I*^2^ larger than 50% indicate the presence of heterogeneity between the studies in analysis [21]. Publication bias was assessed by performing the rank correlation test–Begg’s test [22].

Data analysis was performed in R: a language and environment for statistical computing, version 4.0.3 [23] by using the “metafor” package [24]. Data and code to replicate the analysis are available here: https://github.com/cstrozza91/Meta-Analysis-PANSS-2021.git.

## Results

### Study selection

**Fig. 1:**
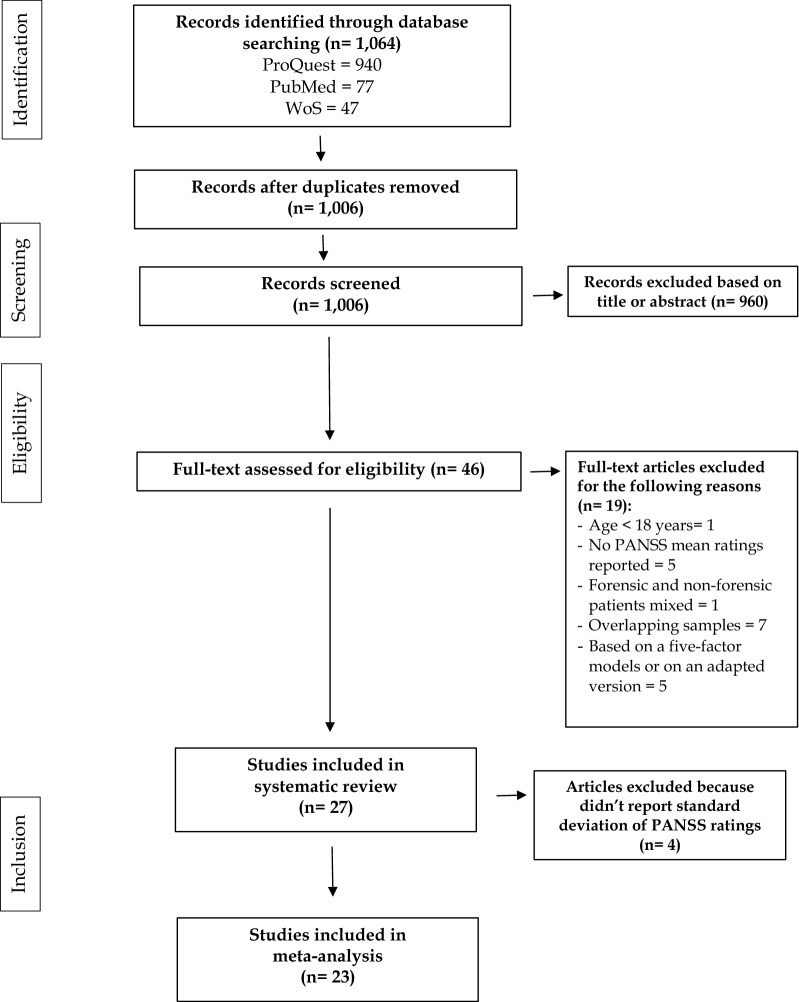
Flow chart of the selection process. PRISMA Preferred Reporting Items for Systematic Reviews and Meta-Analyses

As shown in Fig. 1, out of 1064 articles generated by the preliminary search strategy, 58 were duplicates, and 960 were excluded based on title and abstract as they were irrelevant to study criteria. After reading the full text, a further 19 studies were excluded because they did not report PANSS mean ratings, or because they were based on the five-factor model of the PANSS scoring or on an adapted version of the scale. Several analytic factor solutions have been published and generally supported the presence of five different symptom dimensions: Positive, Negative, Disorganization (often termed “Cognitive”), Affect (often termed “Depression-Anxiety”), and Resistance or Excitement/Activity [25]. However, these studies have shown that the exact composition of the items defining these factors varied [26, 27]. Despite the general similarity of these five-factor models, no single model has achieved broad consensus, and for this reason it is difficult to compare the results of different studies. Therefore, in this systematic review we included only those studies using the original standard PANSS scoring model [10]. This scoring model provides the following scales: Positive, Negative, General, and a Total rating computed using all the 30 items.

Others studies were excluded because they did not report distinct PANSS mean ratings for forensic and non-forensic patients or because they included subjects under 18 years. Finally, when two or more studies reported overlapping samples, priority was given to the study with the largest sample size or to randomized clinical trial (RCT). Finally, 27 studies were included in the systematic review. Of these 27 studies, 4 were excluded from the meta-analytical calculations because they did not report the standard deviations of PANSS ratings, while 23 were used for the meta-analysis: 18 for the Positive scale, 17 for the Negative scale, 15 for General scale, and 17 for the Total rating.

### Studies and sample characteristics

Table 1 shows the characteristics of the 27 included studies. Publications were from 13 countries: Brazil, Croatia, Germany, Ireland, Israel, Japan, Kosovo, the Netherlands, Norway, Turkey, UK, USA, and Russia, with Ireland the country with the largest number of studies (*N* = 9). Studies varied in their sample sizes (from a low of 16 up to 150 forensic patients) and included a total of 1702 participants, most commonly male and inpatients. Sample mean age was 38.4 (SD = 9.6). All studies had samples that included at least 82% of patients with SSD (19 studies 100%), except one study with a percentage of 73.9%. Illness duration and length of stay were reported in only few studies. Regarding PANSS ratings, only 14 studies reported all 4 scales’ ratings: Positive, Negative, General, and Total. In 11 studies there were info about the specific training in PANSS administration received by the assessors [31, 33, 34, 36, 38, 42, 44, 45, 50, 52, 53].

**Table 1:** Characteristic of the 27 studies included in the systematic review and meta-analysis

Authors	Study type	Diagnosis SSD (%)	Forensic sample *N* (%male) MA (sd)	Setting	Illness duration (years) Mean (sd)	Length of stay (months) Mean (sd)	PANSS ratings in forensic patients				Non-forensic sample *N* (%male) MA (sd)	PANSS ratings in non-forensic patients				Quality
							Positive Mean (sd)	Negative Mean (sd)	General Mean (sd)	Total Mean (sd)		Positive Mean (sd)	Negative Mean (sd)	General Mean (sd)	Total Mean (sd)	
*Buckley et al. [45] USA	Cross-sectional	100%	115 (81.7%) 36.6 (10.4)	Mix	14.2 (9.8)	NA	21.2 (8.1)	17.2 (7.2)	34.9 (10.3)	NA	111 (76%) 41.8 (11.7)	15.9 (6.0)	17.0 (7.1)	29.0 (8.0)	NA	High
*Cullen et al. [34] UK	RCT	100%	44 (100%) 35.4 (11.4) 40 (100%) 35.4 (8.4)	In	NA	NA	12.1 (4.4) 10.6 (4.1)	14.2 (5.2) 14.5 (5.4)	NA NA	NA NA	–	–	–	–	–	High
Davoren et al. [58] Ireland	Cohort	82%	86 (100%) 40.6 (12.8) On total sample	In	NA	91.2 On total sample	*N* = 6; 20.3 *N* = 10; 13.6 *N* = 14; 19.2 *N* = 14; 13.0 *N* = 10; 14.6 *N* = 15; 11.5 *N* = 11; 9.5 *N* = 6; 9.3	23.8 22.3 22.2 15.4 21.1 17.1 11.8 11.0	36.3 31.5 33.7 28.4 29.1 26.3 22.8 22.7	80.5 67.4 75.1 56.7 64.8 54.9 44.1 43.0	–	–	–	–	–	High
*Demirbuga et al. [41] Turkey	Cross-sectional	100%	41 (85.4%) 39.0 (8.4)	Out	NA	NA	22.1 (7.4)	18.5 (6.5)	35.6 (6.7)	76.5 (14.4)	35 (65.7%) 39.1 (10.7)	14.9 (7.4)	14.9 (5.6)	28.2 (10.4)	58.0 (20.6)	High
*Donnelly et al. [46] Ireland	Cohort	84%	75 (NA) 41.7 (12.1) On total sample	In	NA	81.6 On total sample	*N* = 17; 15.5 (6.9) *N* = 58; 11.5 (5.0)	NA	NA	62.5 (20.9) 50.2 (16.6)	–	–	–	–	–	High
*Dornan et al. [47] Ireland	Cohort	89.2%	37 (91.8%) 32.3 (NA)	In	NA	26.8	15.7 (8.1)	20.1 (6.9)	34.5 (7.9)	70.4 (17.3)	–	–	–	–	–	High
*Engelstad et al. [36] Norway	Cross-sectional	100%	26 (96%) 38.2 (7.3)	Mix	15.7 (6.7)	NA	11.3 (4.3)	13.0 (5.3)	25.4 (6.0)	48.6 (12.9)	28 (89%) 36.7 (10.1)	10.0 (3.4)	10.5 (3.1)	23.4 (4.3)	43.9 (7.0)	Low
*Frommann et al. [42] Germany	Cross-sectional	100%	19 (100%) 35.3 (8.2)	In	9.4 (8.8)	NA	13.4 (4.0)	15.3 (6.2)	27.9 (6.2)	56.7 (14.6)	19 (100%) 34.7 (10.6)	12.8 (3.4)	14.9 (5.5)	29.0 (5.7)	56.8 (12.3)	Low
*Hornsveld and Nijman [33] Netherlands	Case–control	100%	16 (100%) 33.0 (5.2)	In	NA	20.4	9.9 (1.9)	13.6 (2.5)	26.9 (3.9)	50.4 (4.5)	–	–	–	–	–	High
*Horvath et al. [51] Germany	Cross-sectional	100%	56 (87.5%) 38.5 (10.5)	In	NA	26.5	NA	NA	NA	44.4 (10.9)	–	–	–	–	–	High
*Hundozi et al. [37] Kosovo	Prospective randomized	100%	65 (100%) 39.7 (8.7)	In	15.5 (10.4)	NA	NA	NA	NA	93.4 (8.6)	–	–	–	–	–	Low
*Ivgi et al. [54] Israel	Cohort	100%	150 (100%) 37.2 (10.7)	In	NA	21.7	11.7 (10.7)	15.0 (10.6)	23.2 (14.6)	49.9 (31.5)	–	–	–	–	–	High
*Kashiwagi et al. [38] Japan	Case–control	100%	30 (100%) 44.1 (11.5)	In	18.0 (12.6)	10.8	17.0 (5.6)	20.2 (7.1)	36.8 (9.9)	NA	24 (100%) 40.3 (10.7)	16.9 (7.1)	18.4 (6.5)	32.9 (11.5)	NA	High
Kennedy et al. [48] Ireland	Cohort	73.9%	88 (91%) NA	In	NA	NA	19.3	20.0	37.9	77.0	–	–	–	–	–	High
*Margetić et al. [44] Croatia	Cross-sectional	100%	62 (100%) 43.2 (10.8)	In	NA	72.4	21.1 (5.0)	22.8 (4.9)	45.3 (6.0)	89.3 (13.5)	–	–	–	–	–	High
*Margetić et al. [53] Croatia	Cross-sectional	100%	71 (100%) 43.6 (8.5)	Mix	NA	78	NA	NA	NA	84.4 (15.7)	–	–	–	–	–	High
*Naughton et al. [32] Ireland	Cohort	100% 91%	8 (100%) 35.6 (11.2) 11 (100%) 37.5 (10.6)	In	NA NA	48 44	14.0 (6.3) 11.4 (3.7)	17.5 (4.7) 17.7 (6.7)	27.0 (6.6) 31.7 (8.6)	58.8 (14.9) 60.7 (15.2)	–	–	–	–	–	High
*Nishinaka et al. [39] Japan	Case–control	98.6%	71 (84.5%) 42.8 (11.9)	In	18.1 (9.9)	NA	NA	NA	NA	56.8 (19.6)	–	–	–	–	–	High
*O’Reilly et al. [43] Ireland	Cohort	100%	10 (NA) 36.1 (9.4) 79 (NA) 40.9 (12.7) Male 94.4% On total sample	In	NA NA	36 96	21.6 (8.7) 13.7 (7.0)	25.0 (6.5) 18.9 (7.9)	43.6 (10.4) 29.2 (10.3)	90.1 (19.4) 62.5 (20.0)	–	–	–	–	–	High
*Pillay et al. [57] Ireland	Cross-sectional	85.7%	70 (100%) 42.6 (13.3)	In	NA	111.6	12.8 (6.9)	15.7 (8.3)	29.3 (12.1)	57.8 (24.8)	–	–	–	–	–	High
*Richter et al. [35] Ireland	Cohort	100%	69 (NA) 39.7 (11.1)	In	NA	95.3	14.3 (8.0)	19.1 (7.9)	NA	64.3 (21.8)	–	–	–	–	–	High
Rutledge et al. [49] Ireland	Cross-sectional	88.2% On total sample	102 (91.2%) 38.1 (NA) On total sample	In	NA	NA	*N* = 75; 13.7 *N* = 27; 23.7	20.9 27.8	32.2 42.6	66.7 90.1	–	–	–	–	–	High
*Storozheva et al. [52] Russia	Cross-sectional	100%	28 (100%) 35.3 (2.5)	In	NA	NA	NA	NA	NA	86.8 (2.3)	–	–	–	–	–	High
*Taylor et al. [40] UK	RCT	100%	21 (100%) 40.7 (10.3) 15 (100%) 39.2 (10.6)	In	NA	NA	10.3 (2.8) 10.8 (4.9)	9.7 (2.0) 10.4 (3.1)	24.0 (7.1) 23.4 (7.5)	NA NA	–	–	–	–	–	High
*Teixeira et al. [55] Brazil	Case–control	100%	30 (100%) 38.0 (9.5)	In	15.8	NA	18.7 (6.5)	17.3 (6.6)	35.7 (9.4)	NA	30 (100%) 38.9 (9.5)	18.5 (6.3)	15.8 (5.2)	34.3 (8.6)	NA	High
*Vasic et al. [50] Germany	Cross-sectional	100%	29 (100%) 41.0 (9.3)	In	13.6	NA	14.1 (7.2)	16.2 (6.2)	30.3 (7.6)	NA	31 (100%) 39.0 (12.2)	11.9 (3.9)	13.1 (4.5)	27.0 (6.6)	NA	High
Vinokur et al. [56] Israel	Cohort	100%	60 (90%) 36.1 (9.3) 78 (89.7%) 19.9 (3.3)	In	NA NA	NA NA	17.1 18.4	19.6 16.9	33.4 34.1	70.2 69.4		NA	NA	NA	NA	High

*SSD* schizophrenia spectrum disorders, *MA* mean age, *NA* not available, *In* inpatients, *Out* outpatients, *Mix* mixed setting

^*^Study included in the meta-analysis

Most studies were aimed at assessing the efficacy of some programmes to reduce violence, such as metacognitive training [28], and other psychosocial and rehabilitation interventions [29–31]. Other studies were aimed at investigating neurocognitive functions [32–34], neuropsychological impairment [35], facial emotion recognition, neurocognition, and social cognition [36–39]. Further studies were aimed to investigate patients’ opinions on certain aspects of perceived stigmatization [40], insight deficits [41], working alliance and interpersonal trust in clinicians [42], and decision-making ability [43–45]. Additional studies were centred on psychopharmacological treatment [46, 47] or schizophrenia biomarkers [48]. Finally, one study focused on temperament and character [49], one on a validation tool [50], one on the relationships between delusions and violence [51], one on age of onset and violence [52], one on risk stratification and the care pathway [53], and one on new structured professional judgment instruments for assessing need for therapeutic security, treatment completion, and recovery in forensic settings [54].

### Methodological quality and risk of bias within studies

Among the studies included in this review, there were 3 RCTs [30, 33, 36] and 24 observational studies [28, 29, 31, 32, 34, 35, 37–54]. Risk bias of randomized studies based on the Cochrane quality assessment tool was low (Additional file 2: Table S1). Only one study had four domains with a high risk [33], showing therefore a low quality. All cohort and case–control studies showed a high quality. With respect to cross-sectional studies only two articles were categorized of low quality [32, 38] (Additional files 3: Table S2 and 4: Table S3).

In the seven studies comparing forensic and non-forensic patients, Frommann et al. [38] reported that subjects were matched for age, intelligence, additional addiction, medication and illness duration; in another study [34] forensic and non-forensic subjects were matched for age. In the remaining five studies there is no clear indication of matching, although in all these studies there was a comparison of basic sociodemographic and clinical features, with tests of significance.

### Results of the meta-analysis

**Fig. 2:**
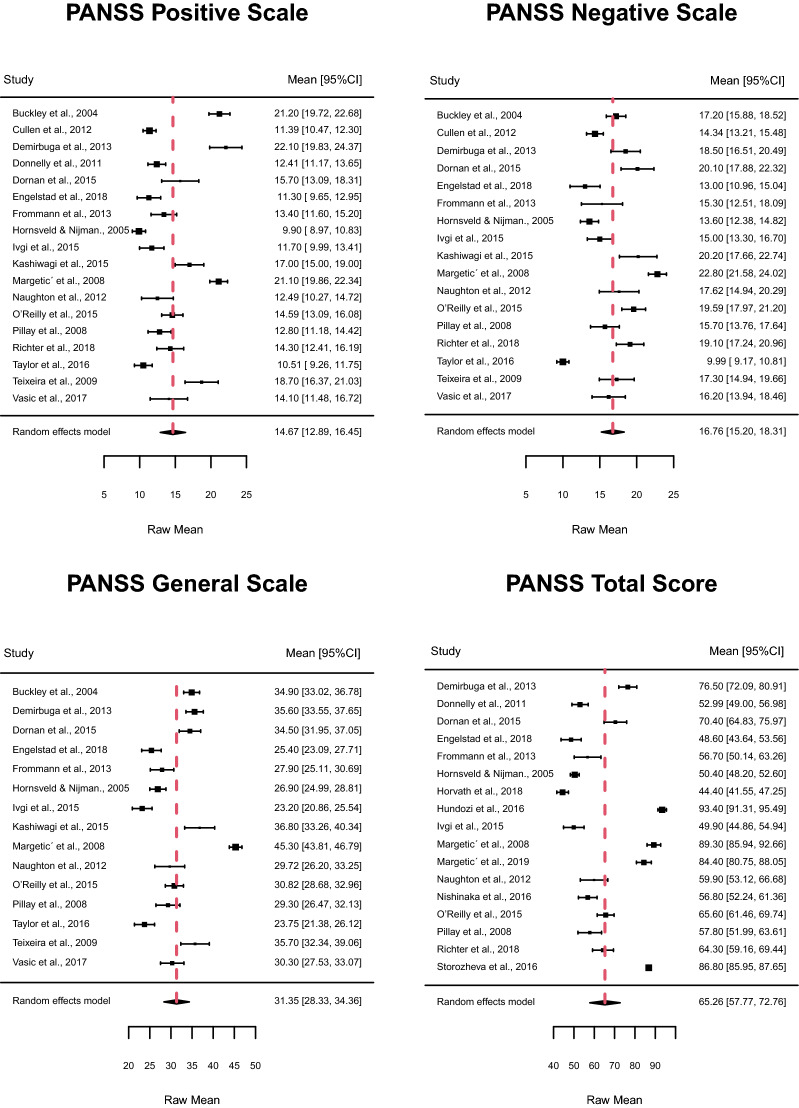
Estimated mean values of PANSS scales in forensic patients

Table 2 (the first line called Total for each scale) shows the estimated mean values of the four PANSS scales for the 23 studies included in the meta-analysis: Positive, Negative, General, and Total. The analysis showed significant *I*^2^ and *Q* ratings (with an overall number of studies considered to be greater than 15), indicating a high level of between-study heterogeneity in terms of symptomatology in forensic patients with SSDs, assessed by the four PANSS scales. Figure 2 shows the estimated mean values, using the random-effects model, for each PANSS scale. Forensic patients’ PANSS mean ratings were as follows: Positive 14.6, Negative 16.7, General 31.3, and Total 65.2. Moreover, only seven studies had a control group of non-forensic patients, and their PANSS mean ratings were as follows: Positive 14.4, Negative 14.9, General 29.1, and Total 52.9.

**Table 2:** Subgroup analysis for mean PANSS ratings

Study subgroups	*N* of studies	Mean ratings				Heterogeneity				Publication bias	
		Estimate	CI 95%		*P*-value	*I*^2^ (%)	Group heterogeneity			Begg’s test	
			Lower limit	Upper limit			*Q*	*df* (Q)	*P*-value	Tau	*P*-value
Positive Scale											
Total	18	14.7	12.9	16.5	0.00	95.8%	453.5	17	0.00	0.3	0.05
SSD %											
100	14	15.0	12.8	17.2	0.00	96.7%	443.7	13	0.00	0.3	0.10
< 100	4	12.9	11.9	14.0	0.00	26.3%	5.1	3	0.16	0.6	0.33
Male %											
100	11	13.8	11.7	16.0	0.00	95.6%	274.4	10	0.00	0.4	0.06
< 100	5	16.9	12.9	20.9	0.00	95.9%	106.5	4	0.00	−0.2	0.82
Mean age											
≥ 36.6	13	14.8	12.7	16.8	0.00	94.7%	252.5	12	0.00	0.3	0.16
< 36.6	5	14.2	10.3	18.2	0.00	97.7%	174.9	4	0.00	0.4	0.48
Negative Scale											
Total	17	16.8	15.2	18.3	0.00	93.6%	418.6	16	0.00	0.1	0.54
SSD %											
100	14	16.5	14.7	18.3	0.00	94.8%	399.6	13	0.00	0.1	0.59
< 100	3	17.7	15.1	20.3	0.00	74.7%	8.5	2	0.01	0.3	1.00
Male %											
100	11	16.1	14.0	18.2	0.00	94.8%	329.5	10	0.00	0.4	0.09
< 100	5	17.6	15.2	20.1	0.00	89.2%	31.3	4	0.00	0.0	1.00
Mean age											
≥ 36.6	13	17.0	15.2	18.9	0.00	93.8%	386.6	12	0.00	−0.0	0.95
< 36.6	4	15.7	12.8	18.5	0.00	91.7%	26.2	3	0.00	0.3	0.75
General Scale											
Total	15	31.4	28.3	34.4	0.00	95.9%	513.6	14	0.00	−0.0	0.92
SSD %											
100	12	31.3	27.6	35.1	0.00	96.9%	503.7	11	0.00	−0.1	0.55
< 100	3	31.2	27.8	34.6	0.00	74.9%	8.6	2	0.01	−0.3	1.00
Male %											
100	10	30.8	26.6	35.1	0.00	96.5%	457.5	9	0.00	0.3	0.16
< 100	5	32.2	28.5	35.9	0.00	93.2%	56.1	4	0.00	−0.4	0.48
Mean age											
≥ 36.6	11	31.4	27.5	35.3	0.00	96.5%	458.6	10	0.00	0.0	1.00
< 36.6	4	31.0	26.8	35.2	0.00	92.8%	46.1	3	0.00	−0.3	0.75
Total scale											
Total	17	65.3	57.8	72.8	0.00	99.0%	2478.9	16	0.00	−0.1	0.60
SSD %											
100	12	67.5	57.4	77.7	0.00	99.4%	2147.6	11	0.00	−0.2	0.25
< 100	5	59.4	53.6	65.2	0.00	84.0%	25.5	4	0.00	0.6	0.23
Male %											
100	9	69.9	58.0	81.8	0.00	99.4	1364.7	8	0.00	−0.3	0.26
< 100	6	60.3	50.2	70.4	0.00	97.0%	199.7	5	0.00	0.2	0.72
Mean age											
≥ 36.6	12	65.3	55.8	74.9	0.00	98.6%	1239.9	11	0.00	−0.2	0.38
< 36.6	5	64.9	52.1	77.7	0.00	98.9%	1021.3	4	0.00	−0.2	0.82

The variables considered in the subgroup analysis were the percentage of patients with SSDs, the percentage of males in the total sample, the setting (inpatients versus outpatients), the mean age and the study quality, assessed by the Newcastle–Ottawa Scale (NOS), and the Cochrane quality assessment tool. Results about the setting and study quality were not included due to the small number of studies related to one of the two subgroups created (inpatients versus outpatients and high versus low study quality). The only statistically significant difference about gender was in the Positive scale: studies including only males reported significantly lower ratings than studies with mixed samples (males and females). Furthermore, the *I*^2^ was greater than 95% demonstrating high heterogeneity between the studies analysed.

**Fig. 3:**
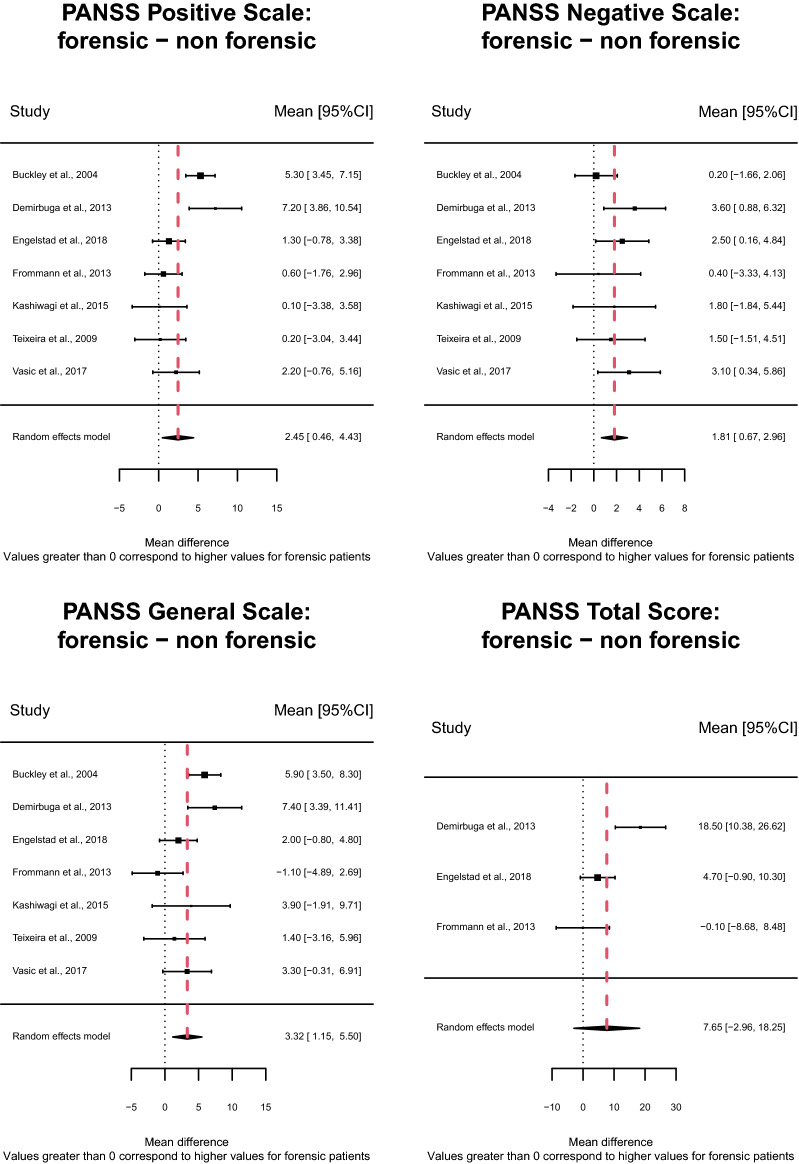
Estimated mean difference between forensic and non-forensic patients

As regards the comparison between forensic and non-forensic patients, significant differences were found in the mean PANSS scale ratings, as suggested by the *p*-values with the relative estimate mean difference, except for the “Total scale” for which the number of studies considered was limited (Table 3). Forensic patients showed higher values in Positive, Negative, and General scales, but the analysis about the Negative scale reported non-significant *I*^2^ and *Q* indicating little between-study heterogeneity in terms of negative symptomatology. This was probably due to the limited number of studies analysed (*N* = 7). Figure 3 shows the estimated mean difference between forensic and non-forensic patients, using the random-effects model, for each PANSS scale.

**Table 3:** Forensic versus non-forensic mean difference PANSS ratings

PANSS scale	*N* of studies	Mean difference				Heterogeneity				Publication bias	
		Estimate	CI 95 %		*P*-value	*I*^2^ (%)	Group heterogeneity			Begg’s test	
			Lower limit	Upper limit			*Q*	*df* (Q)	*P*-value	Tau	*P*-value
Positive	7	2.5	0.5	4.4	0.02	74.8%	23.8	6	0.00	−0.1	0.77
Negative	7	1.8	0.7	2.9	0.00	20.0%	6.2	6	0.39	−0.1	0.77
General	7	3.3	1.2	5.5	0.00	60.2%	15.0	6	0.02	−0.0	1.00
Total	3	7.7	− 3.0	18.3	0.16	83.8%	10.9	2	0.00	−0.3	1.00

## Discussion

This is the first meta-analysis about the severity of psychopathology as assessed with the PANSS in forensic patients with SSD. Our results show that the studies included in this meta-analysis provide a good picture of the level and severity of the overall psychotic symptomatology to be found among forensic patients with SSD, assessed with the PANSS.

Our data show that forensic patients exhibit a mild psychopathological symptomatology. Leucht et al. [55] have speculated on the clinical meaning of PANSS total ratings and have tried to anchor PANSS ratings to CGI ratings in a large sample of patients with schizophrenia. In this way they have identified the following PANSS levels: ‘normal’ (range < 48), ‘borderline mentally ill’ (range 48–60), ‘mildly ill’ (range 61–78), ‘moderately ill’ (range 79–95), ‘markedly ill’, ‘severely ill’ (range 96–118), and ‘extremely ill’ (range > 118) (Additional file 5: Table S4). Based on these data, the mean Total rating of forensic patients included in our meta-analysis points to a clinical condition that can be labelled as ‘mildly ill’. This would hold true only if ratings on each item were broadly similar, for example, with evidence for a high degree of internal consistency for all items contributing to the total rating. This conflicts with the separation between the scales for Positive, Negative, and General symptoms. The use by Leucht et al. [55] of percentage change in the CGI is also problematic as this may not relate meaningfully to baseline severity of symptoms or of functional impairment, though there is some evidence for this [56].

This result may also suggest that a large proportion of these patients were in remission, where “remission” was defined as a low-mild symptom intensity level [57, 58]. Andreasen et al. [57] set a rigorous standard for response (rating of 3 or less in eight key items P1, P2, P3, N1, N4, N6, G5, and G9) and remission, the same sub-threshold ratings in all eight key items sustained for at least 6 months. Others have used much shorter time periods to define response and remission [59]. It is therefore problematic to define a cut-off based on total ratings of a rating scale, under which a patient is considered to be in remission. Schizophrenia is a heterogeneous disorder characterized by a range of possible different symptoms, especially positive and negative symptoms, and their prevalence also depends on the disorder stage [60]. Schizophrenia can be staged as it develops [61]. Positive symptoms have been regarded as features of early and acute schizophrenia, with negative symptoms developing during later stages of the illness [62].

It is worth noting that our results are very different from data obtained in other studies reporting PANSS values in non-forensic patients with schizophrenia: a recent meta-analysis of Matsusaki et al. [63], including 47 trials comparing antipsychotic medications to placebo found much higher PANSS ratings, ranging from a low of 57.6 to a high of 100.8 with a mean of 92.5 (SD = 7.6). A systematic PubMed advanced search [64] reported much higher PANSS ratings than ours, but many clinical characteristics of patients and the illness phase were not clearly specified. They also excluded studies on patients with schizophrenia in remission, following Andreasen’s criteria [57]. Also, in a recent RCT to evaluate the efficacy and safety of a new compound in adults with an acute exacerbation of schizophrenia, the mean Total rating on the PANSS at baseline was much higher as compared to the mean rating found in our forensic sample [65].

### Clinical characteristics of patients studied in forensic settings

Our finding of low PANSS ratings might be explained by sample composition, that is, inpatients living in forensic facilities; these patients may also include patients who were admitted to forensic settings for reasons different from violent offences; while it is impossible to make this discrimination in the selected articles, it is well known that the large majority of people admitted to forensic settings has been admitted specifically because they committed violent offences. Being in a forensic facility implies that patients had guaranteed pharmacological treatment, which promotes clinical stabilization, symptoms reduction, and a better course of the disorder. Moreover, some studies have shown that ensuring treatment compliance decreases the risk of violence [66–68].

Nevertheless, many clinical characteristics of patients included in our meta-analysis were unspecified, and this makes it difficult to draw precise conclusions. For example, illness duration may strongly affect clinical assessment in people with SSD. As suggested by Fountoulakis et al. [69], PANSS ratings probably change according to the stage of schizophrenia. Similarly, Zhao et al. [70] have investigated different stages of schizophrenia comparing first episode to chronic patients. The PANSS total rating clearly indicated that in patients with chronic conditions the severity of symptomatology was lower. In fact, while first-episode patients reported a higher PANSS total rating corresponding to ‘moderately ill’ level, chronic patients showed a lower rating corresponding to ‘mildly ill’ level [55]. This result is consistent with our data.

Interestingly, we found that males had lower ratings than mixed samples on the Positive scale, and this may indicate that female forensic patients exhibit more severe psychotic symptoms. Nevertheless, this result should be interpreted with caution, as the percentage of female patients was very low in all mixed-sample studies (below 20%). In international prison surveys women make up only 10–15% of prison populations in cross-sectional or incident samples [71]; population-based surveys of community violence give a more complex picture in which violence is still less common than in men but less often prosecuted [72–74]. There are population differences between men and women in violence rates, including intimate partner violence involving not only both mental illnesses and personality traits, such as affective instability [73], but also differential social processes in the criminal justice system. A further exploration of this may be beyond the scope of this review.

### Forensic and non-forensic patients with schizophrenia

Another important result of our meta-analysis has to do with the difference between forensic and non-forensic patients: forensic patients had higher ratings than non-forensic patients in all four PANSS scales. While the total rating of forensic patients shows a clinical condition corresponding to ‘mildly ill’ [55], the total rating of non-forensic patients corresponds to ‘borderline mentally ill’. It is important to note that only 7 out of 27 studies included in this meta-analysis reported non-forensic patients. Therefore, the small number of studies does not allow a reliable generalization of this result. Nevertheless, it seems possible to consider that both forensic and non-forensic patients included in these studies were in symptom remission.

Schizophrenia is both a relapsing and remitting illness and a progressive illness that can be staged from prodromal or ‘at risk mental states’ (attenuated psychosis, brief limited intermittent psychosis) with diagnostic criteria that include symptom rating items very similar to PANSS items [75, 76]. These prodromal or ‘at risk’ states can be identified in juvenile forensic populations [77] and may lend themselves to transdiagnostic formulations [78, 79]. Schizophrenia appears to progress through stages from relapsing and remitting to chronic and disabling, and it is unclear whether specific symptom patterns distinguish early stages of illness when violence may be more common [5, 80, 81].

### Outcome evaluations in forensic settings

The use of the PANSS to assess the working of a model of care in forensic psychiatry, such as stratified therapeutic security, is well documented and necessary for the delivery of treatment for violent patients with SSD in forensic hospital and community settings [53, 82]. A prospective cross-validation study indicated that PANSS symptom severity correlates with measures of violence proneness, such as the HCR-20 (Assessing Risk of Violence) dynamic scales, and is a medium-term predictor of inpatient violence and self-harm [83]. Treatment response and completion can be related to reductions in violence proneness, although the explanatory model is complex and includes symptom severity (PANSS) and functional neurocognition [31]. Change in symptom severity measured by PANSS is increasingly recognized as just one measure of therapeutic process and outcome for a forensic model of care, one of the four recoveries: forensic recovery, functional recovery, symptomatic recovery and personal recovery [84].

Measures of personal recovery, such as working alliance and interpersonal trust in physician, are partly confounded by symptom severity [85], as is perceived procedural justice and perceived impact of a review board hearing [42]. Measures of subjective experience and appraisals as part of personal recovery should always be adjusted for symptom severity to take account of subjective perceptual bias due to symptoms.

New models of analysis have allowed an understanding of the relationship between symptoms, such as delusions measured with the PANSS, and outcomes, such as violent acts, when causal modelling includes proximity in time and mediation via symptoms such as anger [8, 13, 86]. This should lend a new and valuable function for PANSS items and ratings in repeated measures paradigms of longitudinal research into causes, for example, concerning the relationship between delusions, moral sentiment, and violent acts [87].

### Limitations

This review has several limitations. First of all, among 27 studies included in this review, only 3 used random samples. Secondly, many studies had very small samples, which reduces the generalizability of our results. Furthermore, the sample is partially heterogeneous with regard to the diagnosis; however, the majority of the sample analysed had a SSD diagnosis: out of 27 studies, only in one case there was a percentage of 73.9 patients with SSD in the study sample, in 5 studies this percentage exceeded 80%, in 2 studies over 90% while 19 studies report 100% of patients with SSD. Our choice was made to include a larger number of studies (as the literature on this topic is rather limited). Moreover, this meta-analysis included mainly male patients and gender differences must be interpreted with caution. The higher percentage of males than females in the study samples is due to the fact that all studies show that males are more frequently violent than females [88, 89]. Besides, these studies often missed information on how much time passed since the index violence to the admission to a forensic institution: PANSS assessment was done at different times, rather than at the time of the relevant violent offence. This fact makes it difficult to clearly understand the association between psychotic symptoms as picked up by PANSS and violent behaviour when it occurred. Furthermore, the selected studies involved patients who signed an informed consent: thus, some forensic patients who were too symptomatic to be able to understand the information required for informed consent were not studied. The results therefore may not completely represent the population of forensic patients. Finally, since forensic patients follow different care pathways in different countries, it is difficult to draw generalizations given the heterogeneity of the population.

## Conclusion

This meta-analysis demonstrates that forensic patients included in studies employing the PANSS for the assessment of psychotic symptoms exhibit a mild symptom severity and therefore may be considered as patients in remission. This result is most likely related to the high compliance to the pharmacological treatments ensured in forensic facilities. This may suggest that treatment is effective in fostering symptom remission even among patients with schizophrenia who committed severe violent acts.

However, it is important to consider that studies included in this meta-analysis do not always define what they mean by violence. Forensic patients are a heterogeneous population and violence can occur in different forms (verbal and/or physical aggression and so on). Additionally, these studies did not report when forensic patients committed the index violence and when they were admitted to forensic services: they may have behaved violently many years ago, and patients’ level of aggressive behaviour at the time of PANSS assessment is not specified. For these latter considerations, it is necessary to be very careful when drawing conclusions about the relationship between symptom severity and violence based on these studies. The association between schizophrenia, and in particular psychotic symptoms, and violence is a very complex phenomenon, and still partially unexplained [86, 87, 90]. In fact, there are many factors that act as key mediators between psychotic symptoms and violence, which should be taken into account to explain this association. For example, a large body of research has identified eight central criminogenic risk factors, including antisocial personality, antisocial attitudes, antisocial peers, substance abuse, history of antisocial behaviour, relationship/familial problems, vocational difficulties, and lack of leisure activities [91]. For all these reasons, further studies on this topic are needed.

## Supplementary Information

**Additional file 1. **Methodological quality assessment and risk of bias

**Additional file 2: Table S1.** Cochrane quality assessment tool.

**Additional file 3: Table S2. **NOS Scale for cohort and case-control studies.

**Additional file 4: Table S3.** NOS Scale for cross-sectional studies.

**Additional file 5: Table S4. **Comparison between PANSS total rating and CGI. ratings, and clinical meaning.

## Data Availability

The datasets generated and/or analysed during the current study are available here: https://github.com/cstrozza91/Meta-Analysis-PANSS-2021.git.

## Abbreviations

PANSS: Positive and Negative Syndrome Scale
SSDs: Schizophrenia spectrum disorders
BPRS: Brief Psychiatric Rating Scale
PRISMA: Preferred reporting items for systematic reviews and meta-analyses
OSF: Open Science Foundation
RCT: Randomized controlled trials
NOS: Newcastle–Ottawa Scale
HCR-20: Assessing risk of violence
