# Supplementary material for: Positive and negative syndrome scale in forensic patients with schizophrenia spectrum disorders: a systematic review and meta-analysis
Source: Ann Gen Psychiatry. 2022 Sep 10;21:36. doi: 10.1186/s12991-022-00413-2 (PMC9463849; doi:10.1186/s12991-022-00413-2)
Supplement: Supplementary file 1 — Additional file 1. Methodological quality assessment and risk of bias [file 12991_2022_413_MOESM1_ESM.docx]

**Additional file 1**

**Methodological quality assessment and risk of bias**

Quality assessment of primary studies was done independently by two authors (C.B. and G.S.) and confirmed by a third author (V.C.) by using the Cochrane quality assessment tool for RCTs [1] and the Newcastle–Ottawa Scale (NOS) for non-randomized studies [2].

The Cochrane quality assessment tool is developed to assess quality of RCTs regarding the following domains: selection, performance, detection, attrition, reporting, and other bias. Overall, the tool assesses the presence of seven evaluation criteria as a whole: random sequence generation, allocation concealment, blinding participants and personnel, blinding outcome assessment, incomplete outcome data, selective reporting, other sources of bias. Each criterion is rated as low risk, high risk, or unclear risk of bias. This tool does not have a cut-off, and for this reason we adopted an ad-hoc evaluation considering as of high quality all selected primary studies which presented at least 4 out of 7 criteria as low risk of bias.

The NOS is one of the most well-known scales for assessing quality and risk of bias in observational studies and contains eight items, categorized into three dimensions including selection, comparability, and - depending on the study type - outcome (cohort studies) or exposure (case-control studies). For each item a series of response options is provided. A star system is used to allow a semi-quantitative assessment of study quality, such that the highest quality studies are awarded a maximum of one star for each item with the exception of the item related to comparability that allows the assignment of two stars. The NOS ranges between zero up to nine stars, with studies having equal or less than 4 stars being identified as representing at high risk of bias, meaning a low quality of the study [3]. An adapted form of the Newcastle-Ottawa Scale for cohort studies was used for cross-sectional studies.

1. Higgins JP, Altman DG, Gøtzsche PC, Jüni P, Moher D, Oxman AD, et al. The Cochrane Collaboration's tool for assessing risk of bias in randomised trials. BMJ. 2011; 343**:** d5928. https://doi.org/10.1136/bmj.d5928
2. Wells GA, Shea B, O'Connell D, Peterson J, Welch V, Losos M, et al. The Newcastle-Ottawa Scale (NOS) for assessing the quality if nonrandomized studies in meta-analyses. Available from: URL: http//www.ohri.ca/programs/clinicaLepidemiology/ oxford.htm [cited 2009 Oct 191.
3. Veronese N, Cereda E, Solmi M, Fowler SA, Manzato E, Maggi S, et al. Inverse relationship between body mass index and mortality in older nursing home residents: a meta-analysis of 19,538 elderly subjects. Obes Rev. 2015; 16(11): 1001–1015. https://doi:10.1111/obr.12309
