# Supplementary material for: Positive and negative syndrome scale in forensic patients with schizophrenia spectrum disorders: a systematic review and meta-analysis
Source: Ann Gen Psychiatry. 2022 Sep 10;21:36. doi: 10.1186/s12991-022-00413-2 (PMC9463849; doi:10.1186/s12991-022-00413-2)
Supplement: Supplementary file 2 — Additional file 2: Table S1. Cochrane quality assessment tool. [file 12991_2022_413_MOESM2_ESM.docx]

**Additional file 2**

**Table 1S** Cochrane quality assessment tool

| **Study** | **Selection**  **bias** | | **Performance bias** | **Detection bias** | **Attrition bias** | **Reporting bias** | **Other**  **bias** | **Total** | **Quality** |
| --- | --- | --- | --- | --- | --- | --- | --- | --- | --- |
|  | *Random sequence generation* | *Allocation concealment* | *Blinding participants and personnel* | *Blinding outcome assessment* | *Incomplete outcome data* | *Selective*  *reporting* | *Other sources*  *of bias* | *High on Risk of Bias* |  |
| Cullen *et al*^34^ | Low | Low | High | High | Low | Unclear | Low | 2/7 | High |
| Taylor *et al*^40^ | Low | Unclear | Low | Low | Low | Unclear | Low | 0/7 | High |
| Hundozi *et al*^37^ | High | High | High | High | Low | Unclear | Low | 4/7 | Low |
