# Supplementary material for: Positive and negative syndrome scale in forensic patients with schizophrenia spectrum disorders: a systematic review and meta-analysis
Source: Ann Gen Psychiatry. 2022 Sep 10;21:36. doi: 10.1186/s12991-022-00413-2 (PMC9463849; doi:10.1186/s12991-022-00413-2)
Supplement: Supplementary file 3 — Additional file 3: Table S2. NOS Scale for cohort and case-control studies. [file 12991_2022_413_MOESM3_ESM.docx]

**Additional file 3**

**Table 2S** NOS Scale for cohort and case-control studies

|  | **Selection** | | | | **Comparability** | **Outcome** | | | **Total** | **Quality** |
| --- | --- | --- | --- | --- | --- | --- | --- | --- | --- | --- |
| **Study** | **Item 1** | **Item 2** | **Item 3** | **Item 4** | **Item 1** | **Item 1** | **Item 2** | **Item 3** |  |  |
| Davoren *et al*^58^ | * | * | * | * | ** | * | * | * | **9** | **High** |
| Donnelly *et al*^46^ | * | * | * | * | ** | * | - | * | **8** | **High** |
| Dornan *et al*^47^ | * | * | * | * | * | * | * | * | **8** | **High** |
| Hornsveld and Nijman^33^ | * | - | - | * | * | - | * | * | **5** | **High** |
| Ivgi *et al*^54^ | * | - | * | * | * | * | * | - | **6** | **High** |
| Kashiwagi *et al*^38^ | * | * | * | * | * | * | * | - | **7** | **High** |
| Kennedy *et al*^48^ | * | * | * | * | * | - | - | - | **5** | **High** |
| Naughton *et al*^32^ | - | * | * | * | * | - | * | * | **6** | **High** |
| Nishinaka *et al*^39^ | * | * | * | * | * | * | * | * | **8** | **High** |
| O’Really *et al*^43^ | * | * | * | * | * | * | * | * | **8** | **High** |
| Richter *et al*^35^ | * | * | * | * | * | * | * | * | **8** | **High** |
| Teixeira *et al*^55^ | * | * | - | * | * | * | * | - | **6** | **High** |
| Vinokur *et al*^56^ | * | * | * | * | * | - | - | - | **5** | **High** |

*Note.* A study can be awarded a maximum of one star for each numbered item within the Selection and Outcome categories. A maximum of two stars can be given for Comparability.
