# Supplementary material for: Positive and negative syndrome scale in forensic patients with schizophrenia spectrum disorders: a systematic review and meta-analysis
Source: Ann Gen Psychiatry. 2022 Sep 10;21:36. doi: 10.1186/s12991-022-00413-2 (PMC9463849; doi:10.1186/s12991-022-00413-2)
Supplement: Supplementary file 4 — Additional file 4: Table S3. NOS Scale for cross-sectional studies. [file 12991_2022_413_MOESM4_ESM.docx]

**Additional file 4**

**Table 3S** NOS Scale for cross-sectional studies

|  | **Selection** | | | | **Comparability** | **Outcome** | | **Total** | **Quality** |
| --- | --- | --- | --- | --- | --- | --- | --- | --- | --- |
|  | **Item 1** | **Item 2** | **Item 3** | **Item 4** | **Item 1** | **Item 1** | **Item 2** |  |  |
| Buckley *et al*^45^ | * | * | - | ** | * | - | - | **5** | **High** |
| Demirbuga *et al*^41^ | * | * | - | ** | * | - | - | **5** | **High** |
| Engelstad *et al*^36^ | * | - | - | ** | * | - | - | **4** | **Low** |
| Frommann *et al*^42^ | * | - | - | ** | * | - | - | **4** | **Low** |
| Horvath *et al*^51^ | * | * | - | ** | * | - | - | **5** | **High** |
| Margetić *et al*^44^ | * | * | - | ** | * | - | - | **5** | **High** |
| Margetić *et al*^53^ | * | * | - | ** | * | - | - | **5** | **High** |
| Pillay *et al*^57^ | * | * | - | ** | * | - | * | **6** | **High** |
| Rutledge *et al*^49^ | * | * | - | ** | * | - | - | **5** | **High** |
| Storozheva *et al*^52^ | * | * | - | ** | * | - | - | **5** | **High** |
| Vasic *et al*^50^ | * | * | - | ** | * | - | - | **5** | **High** |

*Note*. A study can be awarded a maximum of one star for each numbered item within the Selection and Outcome categories. A maximum of two stars can be given for Item 4 of Selection and for Comparability.
