# Supplementary material for: Positive and negative syndrome scale in forensic patients with schizophrenia spectrum disorders: a systematic review and meta-analysis
Source: Ann Gen Psychiatry. 2022 Sep 10;21:36. doi: 10.1186/s12991-022-00413-2 (PMC9463849; doi:10.1186/s12991-022-00413-2)
Supplement: Supplementary file 5 — Additional file 5: Table S4. Comparison between PANSS total rating and CGI. ratings, and clinical meaning. [file 12991_2022_413_MOESM5_ESM.docx]

**Additional file 5**

**Table 4S** Comparison between PANSS total rating and CGI ratings, and clinical meaning

| **PANSS TOTAL**  **RATINGS** | **CGI RATINGS** | **CLINICAL MEANING** |
| --- | --- | --- |
| < 48 | 1 | Normal |
| 48-60 | 2 | Borderline mentally ill |
| 61-78 | 3 | Mildly ill |
| 79-95 | 4 | Moderately ill |
| 96-118 | 5 | Markedly ill |
| 119-147 | 6 | Severely ill |
| >147 | 7 | Among most extremely ill patients |

*Note*. Ratings extracted from the article of Leucht *et al*^59^
